# Patient-centered web-based information on oral lichen planus: Quality and readability

**DOI:** 10.4317/medoral.22992

**Published:** 2019-06-28

**Authors:** Alejandro-Ismael Lorenzo-Pouso, Mario Pérez-Sayáns, Omar Kujan, Pablo Castelo-Baz, Cintia Chamorro-Petronacci, Abel García-García, Andrés Blanco-Carrión

**Affiliations:** 1 DDS, MSc, PhD. Oral Medicine, Oral Surgery and Implantology Unit, Faculty of Medicine and Odontology, University of Santiago de Compostela, Santiago de Compostela, Spain; 2 GI-1319 Research Group, Health Research Institute of Santiago de Compostela (IDIS), Santiago de Compostela, Spain; 3 DDS, PhD, UWA Dental School, University of Western Australia, Nedlands WA 6009, Australia; 4 DDS, PhD, Endodontics Unit, Faculty of Medicine and Odontology, University of Santiago de Compostela, Santiago de Compostela, Spain

## Abstract

**Background:**

To assess the readability and quality of web-based information available for patients about oral lichen planus (OLP).

**Material and Methods:**

Three major search engines (Google, Bing and Yahoo!) were used to identify websites of particular interest to the study using the search term ‘oral lichen planus’. The first 100 sites of each search engine were considered for the study. The quality of the contents was evaluated using the DISCERN instrument. The Flesch-Kinkaid Reading Grade Level (FKRGL) and the Flesh Reading Ease Score (FRES) were used to assess readability. The presence of the Health on the Net (HON) seal was also evaluated.

**Results:**

Following the application of the study’s exclusion criteria, only 28 websites were compiled for further analysis. The median of the DISCERN instrument was 3 [2-3] which means serious or potentially important shortcoming in the quality of the information. Readability indexes pointed to a high reading difficulty (FRES: 48.14±11.22; FKRGL:11.13±2.90).

**Conclusions:**

The information provided by the Internet to the general public regarding OLP has major deficits in terms of quality, and at the same time is difficult for a comprehensive reading. Further studies are warranted to test well-produced patient-centered information on OLP.

** Key words:**Oral lichen planus, oral diseases, health literacy, health information.

## Introduction

Lichen planus is a chronic T cells-mediated muco-cutaneous disease of unknown origin (1). Skin and oral mucosa are the most commonly affected areas. It is sometimes accompanied by injuries in other anatomic sites such as the genitals, conjunctiva, scalp or nails (2,3). It affects 0.5-2.2% of the population and is more frequent in women than in men (4). Oral lichen planus (OLP) may present in several forms, such as white stria, plaques, papules and erythematous or erosive lesions (5,6). Given the high prevalence of this pathology, it is the most common non-infectious oral mucosal disease in adult patients referred to dental clinics (4).

A dilemma frequently arises concerning the need of active treatment or the use of a watchful waiting due to OLP likelihood of self-resolution. Several management approaches have been used to control this pathological condition including corticosteroids, retinoids, immunosuppressant (cyclosporine, levamisole, azathioprine and tacrolimus), psoralen with ultraviolet A therapy (PUVA) and, other phytochemicals. When lesions are concentrated in the oral cavity, topical pharmaceuticals are frequently preferred over systemic administration to reduce side effects. The current gold standard treatment for symptomatic OLP is the use of topical corticosteroids (7).

The Internet community is used daily by more than 3,700 million people, which represents a percentage near the half of today’s world population according to Internet world stats (available at: https://www.internetworldstats.com/). A large number of patients are consumers of internet-based health information in the world despite the great limitations observed in many of these data (8). Some studies demonstrated that patients believe that the information they get from the Internet about their pathologies will be equal or even better than the one provided by their health professional (9). At the time health care professionals took notice of these realities, they developed mechanisms to help patients in their search for reliable web-based health information. In terms of search engines, Google holds the majority market share (i.e., 89.2%) according to Statista (available at: https://www.statista.com/statistics/216573/worldwide-market-share-of-search-engines/). Ironically some authors have come up with the term Dr. Google to point out the strong influence exerted by this type of e-health information on patients (10).

Patients suffering from OLP are a particularly vulnerable group because the comorbidity of this disease with the presence of symptoms of depression and anxiety (1). In addition, a relevant fraction of OLP-affected patients develop cancerophobia (11). Oral cancer is the fourth most frequent topic that arises as topic in the dental office after patients searching for health information on the internet (7). It is important to know if the contents on the web relate these two pathologies and see how this relationship is made known for the general public. A recent review (12) estimated and annual malignant transformation rate of 0.2% for OLP and suggested three relevant risk factors: female gender, red clinical forms, and tongue localization on the rationale of Krutchkoff´s criteria (13).

This study aims to assess the quality and readability of the current web-based information for the treatment of patients with OLP using three most common globally used search engines (Google, Bing and Yahoo!).

## Material and Methods

-Search strategy

Three search engines have been used in this study; Google (www.google.com), Bing (www.bing.com) and Yahoo! (www.yahoo.com). The searches were conducted in August 2017, using the term “oral lichen planus” without any filters, and using the English language for the interface and operative system. The websites were compiled on an external hard drive for later analysis. The first 100 websites of each search engine given were considered for the study. Exclusion criteria included irrelevant or inappropriate content, commercial only websites, links to scientific articles of abstracts, duplicate websites, forums, videos, online medical dictionaries and websites with broken links (14).

The Webpages were firstly identified, and then they were classified by specialization (entirely or partially related to OLP treatment) and affiliation (non-profit organ, commercial, or university centres or professional societies). The content type was also classified (medical findings, clinical assays, human interest accounts, questions and answers). Information on the authors of the included websites including their nature of profession were reported. Included websites were then verified by checking for the Health On the Net (HON) seal. This seal establishes the quality, reliability and scientific rigor of the health content published in any Web related to health (available at: https://www.hon.ch).

-Quality assessment

The DISCERN questionnaire is a valid and reliable tool to evaluate the quality of health information (www.discern.org.uk/background) (15). This questionnaire is constituted from 16 items that are subdivided into 3 sections. The first section (items 1-8) is related to the confidence that can be deposited in the source of information, the second section (9-15) refers to the quality of the information in relation to the treatment options, and the last section it only consists of one item (16) that evaluates the overall quality of the information. All these items are based on Likert scales.

The assessment of questionnaire using the Likert scales was performed independently by two experts in oral medicine (ALP and MPS), and in the case of discrepancy, a final decision was achieved by the third assessor (ABC).

-Readability assessment

Readability is the ease with which a reader can understand a written text. The Flesch-Kinkaid Reading Grade Level (FKRGL) and Flesch Reading Ease (FRES) were used to evaluate the readability of the selected sites. These scoring systems are validated to evaluate the readability of information written in English and have already been used in the field of dentistry previously (14). These indexes were calculated with an online tool (www.readabilityformulas.com) from fragments of texts between 300-500 words copied and pasted from each website. It was also decided to measure the accuracy of this online tool with the following formulas: FRES = 206,835 - (1,015 x Average number of words per sentence) - (84.6 x Average number of syllables per word); FKRGL = (0.39 x Average number of words per sentence) + (11.8 x Average number of syllables per word) - 15.59. Readability grades according to the Flesch Reading Ease Score are: 0-30 = very difficult; 30-50 = difficult; 50-60 = fairly difficult; 70-80 = fairly easy; 80-90 = easy; and 90-100 = very easy. Text with “easy” label by the Flesch-Kinkaid Reading Grade level is considered readable by people up to 12 years’ of age; text labelled as “difficult” is suitable for people aged over 16. Websites were also graded according to the FKRGL scale as easy (≤6th grade level) or difficult (≥10th -grade level) to read (14,16).

-Statistical analysis

Frequency analysis was performed for categorical variables and the mean ± standard deviation was calculated for the continuous variable. The Mann-Whitney test was used to compare the variables. The significance value used was taken when the p value was equal to or less than 0.05. The results were analysed using the statistical software package SPSS v 21.0 for Windows (SPSS Inc., Chicago, IL, USA).

## Results

**Figure 1:**
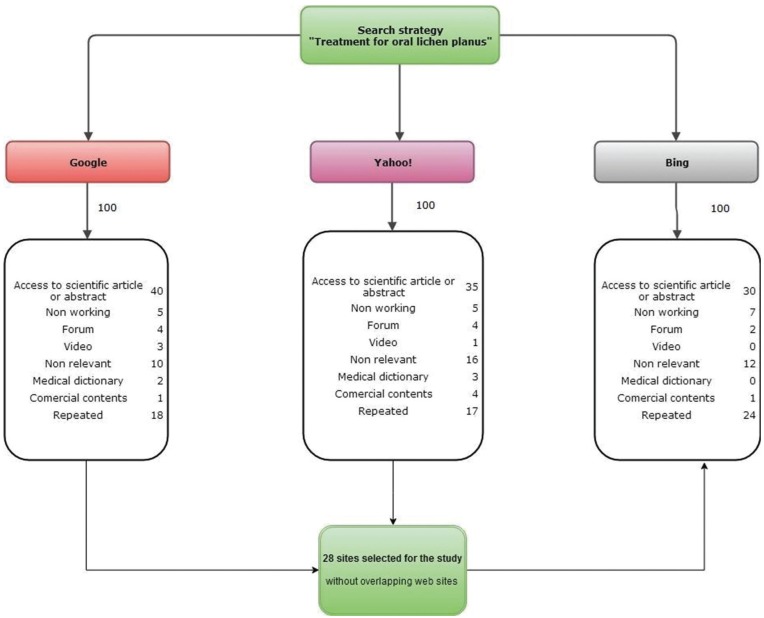
Flow chart of the study.

The initial search yielded 300 results among the search engines. Applying the exclusion criteria 83 Websites were not valid in the Google search, 85 in Yahoo! and 76 in Bing. Then, after deleting overlapping sites only 28 unique sites remained for further analysis (Fig. 1).

In relation to the authorship of the web pages, 20 addresses did not clarify authorship (71.4%), compared to 8 that did (28.6%). Among those who determined the author, half of them were written by journalists and the other half was undertaken by health professionals mainly dermatologists and dentists. Classification of the affiliation of these web addresses counted 12 web pages that belonged to non-profit organizations (42.9%), 9 related to organizations for commercial purposes (32.1%), 3 dependent of medical centres (10.7% %) and 4 dependent of governmental centres (14.3%). Identifying the exclusivity of the content, 16 web addresses (57.1%) were exclusively dedicated to the subject of the study in contrast to 12 (42.9%) who dealt with a bigger number of topics. When the formats of contents were revised they were classified as follows: medical findings 16 (57.1%), the questions and answers 7 (25.0%), human interest accounts 4 (14.3%) and clinical assays 1 (3.6%).

**Figure 2:**
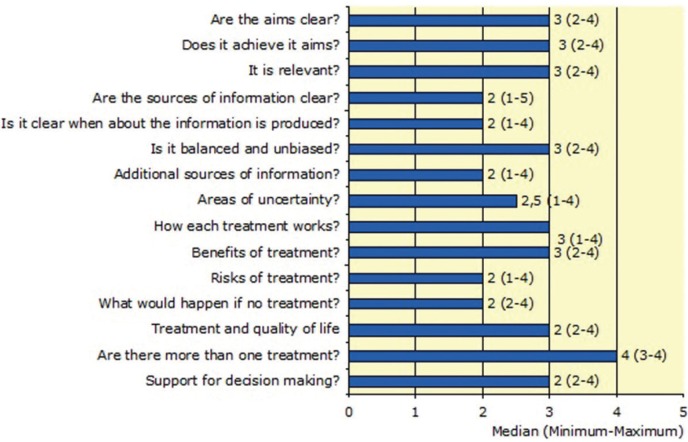
Median quality rating of the 28 included websites according to the DISCERN instrument.

Finally, in this section we value the presence or not of the HON seal in the web pages under analysis. Only 5 Web pages (17.9%) were identified with the seal, compared to 23 (82.1%) that did not contain it. Overall rating (DISCERN; Item 16) of all web pages was 3 with a range of 2-4 so serious or potentially important shortcoming in the quality of the information obtained can be assumed. Figure 2 shows the results about 1-15 DISCERN Items. The variability of the range of these items demonstrates the heterogeneity of the information consulted. No statistically significant correlation between the existence of the HON seal on the web pages and the Overall Rating of DISCERN (Item 16) (*p* = 0.193) was found. Interestingly, a statistically significant association between DISCERN items 4 and 5 concerning incorporating the sources of information and the date of revision with the author’s authorship declaration was found (*p* = 0.02, *p* = 0.01 respectively).

**Figure 3:**
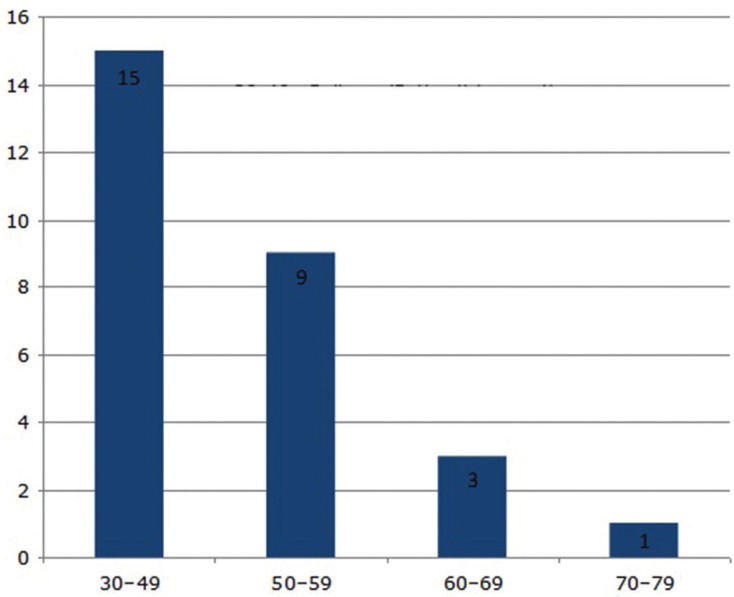
Distribution of FRES scores among included websites. 30-49: college (difficult to read) 50-59: 10th to 12th grade (fairly difficult to read) 60-69: 8th to 9th grade (plain English) 70-79: 7th grade (fairly easy to read)

The readability indexes reached values that denote a high degree of difficulty to reach a comprehensive reading (FRES: 48.14 ± 11.22; FKRGL: 11.13 ± 2.90). Further, Web pages containing the HON seal were found to be not significantly more readable according to both indexes (FRES; *p* = 0.95) (FKRGL; *p* = 0.90). The readability of web directions based on the FRES is shown in Figure 3.

## Discussion

Medicine history has witnessed a turning point during the last decades due to the progressive erosion of the physicians’ paternalistic attitude (17). Nowadays other trends have aroused in the search of upgrading patients’ autonomy and their active participation on treatment decisions. Thus, shared decision making (SDM) is extremely relevant in current clinical practice (18). SDM has proven usefulness in optimizing self-efficacy among patients and it can be influenced by decision support systems such as Internet. In this line, Internet can help to bridge or enlarge the gap between patients and physicians (19). The exploration of the quality of these potential online decision aids has captured the interest of a bulk of research.

When the focus is put web-based information on dentistry and related topics a handful of research can be identified for several issues like: periodontology, implant dentistry, oral mucosa diseases, or oral oncology (20-25). It is difficult to draw a global conclusion of these works because of their methodological variety. Perhaps even despite the disparity the results mostly point to poor quality and legibility in patient-focused web-based health information on the field of dentistry. Specifically regarding OLP internet information both existing papers agree on its poor quality by means of DISCERN rating and JAMA benchmarks (26,27). Nonetheless, to our best knowledge no previous research has focused on elucidate the readability of OLP-related websites.

In the present paper, we set out to determine both quality and readability on OLP-related available web-based information by means of three standardised tools previously validated to evaluate this information. Only 28 pages remained after applying exclusion criteria, and of them only 5 websites had the HON seal; so accurate internet information regarding this matter remains difficult to identify for patients. The majority of the included websites in this research were beyond recommended readability levels so a relevant portion of lay people will find difficulties for its comprehension (14). According to DISCERN tool no website reached a 5-point score in all items. Lopez-Jornet et al. (26) and Hu *et al.* (27) also come to this finding. It is important to be aware that DISCERN is a validated and widely used tool for determining the reliability of medical contents, but it was not originally designed to assess the accuracy of a scientific content displayed on a Website (15).

According to American Medical Association written patient education materials should be 5th or 6th grade levels (28). Our results fall within wide limits, nonetheless, overall they do not follow these recommendations. This may be motivated by the use of complex and technical vocabulary.

Taking account, the frequently altered psychological profile of OLP-affected patients, characterised by somatization, anxiety and/or depression its mandatory to act upon this information (29). Special emphasis should be placed on relevant controversies regarding OLP such its malignant transformation or its relationship with hepatitis virus c infection (5,6). This will prevent the creation of false health clichés and ultimately help to stablish a better patient-doctor relationship. During data collection a handful of contents related with these topics emerged like webpages indicating that all OLP-affected patients must get tested for hepatitis C. We agree with Lodi *et al.* (5) that to perform this test is mandatory in these patients but we believe that clinicians should individualized each case before taking action on this matter and e-health information should be more cautious at the time of supplying this information. Although malignant transformation of OLP is an uncommon feature is one of the most treated issues on OLP-related e-health information and patients might have a harder time deciding if it is real and a matter to worry about. A lot of contents related to pseudoscience solutions for OLP emerged during data collection specially about homeopathy and traditional Chinese medicine.

Incorrect translations can make e-health information more difficult to comprehend and might provide incorrect information to patients, in addition specific oral medicine related terminology can result in misunderstanding and ultimately in a poorer patient-doctor relationship (30). Use of some inappropriate terms in Spanish in oral medicine, especially dispensable anglicisms has been described as a problematic issue (31). Also some terms can per se create hard feeling on patients such as the use of “cyst” as “a type of tumour”, this is due to the fact that the word “tumour” is unconsciously mistaken as a synonymous of ‘malignancy’ by patients (32). It is essential that all website builders appropriately modify language and use comprehensive translations in their contents in order to deliver information in a straightforward manner.

This paper presents some limitations. Especially, web pages are constantly changing, so this work is the reflection of a particular moment. At the same time, this work only retrieved information from three search engines and only English-written contents. Additionally, the present work shows the inherent weaknesses of applied tools. Strengths of the current research is the use of two dimensions (readability and quality) instead of a single one like previous research on OLP web-based contents.

## Conclusions

Patient-centered web-based information about OLP is poor in terms of quality and also difficult to read. The use of these kind of evaluation tools may be of help to optimize or at least take notice of this reality. Medical professionals should help patients in order to achieve an ideal SDM, especially in this subgroup of patients that frequently show a high tendency to somatization.

Future lines of research should contribute towards our findings in order to understand the exact impact of OLP online information in patients´ outcome and SDM. Patients should always remember that the information found online should not take the place of medical advice and clinicians must be aware of the importance of e-health information in their clinical practice.
